# Essential norm of some extensions of the generalized composition operators between *k*th weighted-type spaces

**DOI:** 10.1186/s13660-017-1493-x

**Published:** 2017-09-13

**Authors:** Stevo Stević

**Affiliations:** 1 Mathematical Institute of the Serbian Academy of Sciences, Knez Mihailova 36/III, Beograd, 11000 Serbia; 2 Operator Theory and Applications Research Group, Department of Mathematics, King Abdulaziz University, P.O. Box 80203, Jeddah, 21589 Saudi Arabia

**Keywords:** 47B38, 47B33, 30H99, essential norm, generalized composition operator, *k*th weighted-type space, unit disk

## Abstract

We calculate the essential norm of some extensions of the generalized composition operators between *k*th weighted-type spaces on the unit disk in the complex plane, considerably extending some results in the literature.

## Introduction

Let ${\mathbb {D}}$ be the open unit disk in the complex plane ${\mathbb {C}}$, $H({\mathbb {D}})$ the class of all holomorphic functions on ${\mathbb {D}}$, and $S({\mathbb {D}})$ the class of all holomorphic self-maps of ${\mathbb {D}}$.

Let $\mu(z)$ be a positive continuous function on ${\mathbb {D}}$ (weight) and $k\in {\mathbb {N}}_{0}$. The *k*th weighted-type space denoted by ${\mathcal {W}}^{(k)}_{\mu}({\mathbb {D}})={\mathcal {W}}^{(k)}_{\mu}$ is defined as follows:

$${\mathcal {W}}^{(k)}_{\mu}= \bigl\{ f\in H({\mathbb {D}}): b_{{\mathcal {W}}^{(k)}_{\mu}}(f)< \infty \bigr\} , $$

where

1 $$ b_{{\mathcal {W}}^{(k)}_{\mu}}(f):=\sup_{z\in {\mathbb {D}}}\mu (z)\big|f^{(k)}(z)\big|. $$

The space was introduced in [1] where the composition operators from the weighted Bergman space to the space were studied. Some other concrete operators on the space were later studied in [2–4].

If $k=0$, then $b_{{\mathcal {W}}^{(0)}_{\mu}}(\cdot)$ is a norm on space ${\mathcal {W}}^{(0)}_{\mu}$, the so-called weighted-type space ([5, 6]). If $k\in {\mathbb {N}}$, then it is easy to see that $b_{{\mathcal {W}}^{(k)}_{\mu}}(\cdot)$ is a semi-norm on ${\mathcal {W}}^{(k)}_{\mu}$. It is not a norm on the space since from $b_{{\mathcal {W}}^{(k)}_{\mu}}(f)=0$ it follows that $f^{(k)}(z)=0$, $z\in {\mathbb {D}}$, and consequently $f(z)=p_{k-1}(z)$, where $p_{k-1}$ is a polynomial of degree at most $k-1$. However, it is a norm on the quotient space ${\mathcal {W}}^{(k)}_{\mu}/{\mathbb {P}}_{k-1}$, where ${\mathbb {P}}_{k-1}$ is the space of all polynomials of degree less than or equal $k-1$. Indeed, let $f+{\mathbb {P}}_{k-1}\in{\mathcal {W}}^{(k)}_{\mu}/{\mathbb {P}}_{k-1}$, and, based on the definition of a norm on a quotient space, let

2 $$ \|f+{\mathbb {P}}_{k-1}\|_{{\mathcal {W}}^{(k)}_{\mu}/{\mathbb {P}}_{k-1}}:=\inf_{g\in f+{\mathbb {P}}_{k-1}}b_{{\mathcal {W}}^{(k)}_{\mu}}(g). $$

Then, if $\|f+{\mathbb {P}}_{k-1}\|_{{\mathcal {W}}^{(k)}_{\mu}/{\mathbb {P}}_{k-1}}=0$, by using (1) and (2), we have

$$0=\inf_{g\in f+{\mathbb {P}}_{k-1}}b_{{\mathcal {W}}^{(k)}_{\mu}}(g)=\inf_{p_{k-1}\in {\mathbb {P}}_{k-1}}b_{{\mathcal {W}}^{(k)}_{\mu}}(f+p_{k-1})_{{\mathcal {W}}^{(k)}_{\mu}}=b_{{\mathcal {W}}^{(k)}_{\mu}}(f), $$

from which it follows that $f\in {\mathbb {P}}_{k-1}$, that is, $f+{\mathbb {P}}_{k-1}={\mathbb {P}}_{k-1}=0_{{\mathcal {W}}^{(k)}_{\mu}/{\mathbb {P}}_{k-1}}$.

On the other hand, there are some natural algebraic isomorphisms between some quotient spaces and some spaces of holomorphic functions. Namely, we have

$$H({\mathbb {D}})/{\mathbb {P}}_{k-1}\cong_{a} \bigl\{ f\in H({\mathbb {D}}): f^{(j)}(0)=0, j=\overline {0,k-1} \bigr\} =:H_{k}({\mathbb {D}}), $$

and

$${\mathcal {W}}^{(k)}_{\mu}/{\mathbb {P}}_{k-1} \cong_{a} \bigl\{ f\in{\mathcal {W}}^{(k)}_{\mu}: f^{(j)}(0)=0, j=\overline {0,k-1} \bigr\} =:{\mathcal {W}}^{(k)}_{\mu,k}( {\mathbb {D}}). $$

Indeed, for each class $g+{\mathbb {P}}_{k-1}\in H({\mathbb {D}})/{\mathbb {P}}_{k-1}$ (or $g+{\mathbb {P}}_{k-1}\in{\mathcal {W}}^{(k)}_{\mu}/{\mathbb {P}}_{k-1}$) there is a unique $f_{g}\in g+{\mathbb {P}}_{k-1}$ such that $f^{(j)}_{g}(0)=0$, $j=\overline {0,k-1}$. Namely, if $g(z)=\sum_{j=0}^{\infty}a_{j}z^{j}$, then we can take $f_{g}(z)=\sum_{j=k}^{\infty}a_{j}z^{j}$, that is, $f_{g}=g+p_{g,k-1}$, where $p_{g,k-1}(z)=\sum_{j=0}^{k-1}(-a_{j})z^{j}$, and the map

$$L(g+{\mathbb {P}}_{k-1}):=f_{g} $$

is a linear bijection from $H({\mathbb {D}})/{\mathbb {P}}_{k-1}$ onto $H_{k}({\mathbb {D}})$, as well as from ${\mathcal {W}}^{(k)}_{\mu}/{\mathbb {P}}_{k-1}$ onto ${\mathcal {W}}^{(k)}_{\mu,k}({\mathbb {D}})$. Hence, we can identify the quotient spaces with the corresponding subspaces of holomorphic functions satisfying the conditions $f^{(j)}(0)=0$, $j=\overline {0,k-1}$.

From (1) and (2) it follows that

$$\|f+{\mathbb {P}}_{k-1}\|_{{\mathcal {W}}^{(k)}_{\mu}/{\mathbb {P}}_{k-1}}=b_{{\mathcal {W}}^{(k)}_{\mu}}(f), $$

this fact along with the above mentioned algebraic isomorphism shows that the spaces $({\mathcal {W}}^{(k)}_{\mu}/{\mathbb {P}}_{k-1}, \|\cdot\| _{{\mathcal {W}}^{(k)}_{\mu}/{\mathbb {P}}_{k-1}})$ and $({\mathcal {W}}^{(k)}_{\mu ,k}({\mathbb {D}}), b_{{\mathcal {W}}^{(k)}_{\mu}}(\cdot))$ are isometrically isomorphic, that is, ${\mathcal {W}}^{(k)}_{\mu}/{\mathbb {P}}_{k-1}\cong{\mathcal {W}}^{(k)}_{\mu,k}({\mathbb {D}})$. So, they can be identified, and we can regard it to be the same if we say $f\in {\mathcal {W}}^{(k)}_{\mu}/{\mathbb {P}}_{k-1}$ or $f\in{\mathcal {W}}^{(k)}_{\mu ,k}$.

Let

3 $$ \|f\|_{{\mathcal {W}}^{(k)}_{\mu}}=\sum_{j=0}^{k-1}\big|f^{(j)}(0)\big|+ \sup_{z\in {\mathbb {D}}}\mu(z)\big|f^{(k)}(z)\big|, $$

where *μ* is a weight and $k\in {\mathbb {N}}_{0}$ (for $k=0$ we use the standard convention $\sum_{j=l}^{l-1}a_{j}=0$, $l\in {\mathbb {Z}}$). Then it is easy to see that (3) defines a norm on space ${\mathcal {W}}^{(k)}_{\mu}$, and that $({\mathcal {W}}^{(k)}_{\mu}, \|\cdot\| _{{\mathcal {W}}^{(k)}_{\mu}})$ is a Banach space. The normed space is a natural generalization of the weighted-type, Bloch-type and Zygmund-type spaces (see, *e.g.*, [7–10]).

Let $L:X\to Y$ be a linear bounded operator, that is, it maps bounded sets of *X* into bounded sets of *Y*. By $\|L\|_{X\to Y}$, we denote the operator norm of $L:X\to Y$, that is, $\|L\|_{X\to Y}=\sup_{\|x\| _{X}\le1}\|L(x)\|_{Y}$. An operator $K:X\to Y$ is called compact if it maps bounded subsets of *X* into relatively compact subsets of *Y*.

Essential norm of a bounded operator $L:X\to Y$ is defined by

$$\|L\|_{e, X\to Y}:=\inf_{K\in{\mathcal {K}}(X,Y)}\|L-K\|_{X\to Y}=\inf _{K\in{\mathcal {K}}(X,Y)}\sup_{\|x\|_{X}\le1}\big\| L(x)-K(x)\big\| _{Y}, $$

that is, as the distance of operator *L* to the set of compact operators ${\mathcal {K}}(X,Y)$.

Let

$$(Df) (z)=f'(z) $$

be the standard differentiation operator on $H({\mathbb {D}})$. By $D^{k}$ we will denote the composition of (exactly) *k* differentiation operators, that is, if $f\in H({\mathbb {D}})$, then

$$D^{k} f=\underbrace{D\bigl(D\bigl(\cdots(D}_{k\text{-times}}f)\cdots \bigr)\bigr). $$

Let

4 $$ I_{k}(f) (z):= \int_{0}^{z} \int_{0}^{\zeta_{k}}\cdots \int _{0}^{\zeta_{2}}f(\zeta_{1})\,d \zeta_{1} \,d\zeta_{2}\cdots \,d\zeta_{k}, $$

where $k\in {\mathbb {N}}$ and $f\in H({\mathbb {D}})$.

It is clear that $D^{k}I_{k}f=f$ for every $f\in H({\mathbb {D}})$, that is,

5 $$ D^{k}I_{k}=\mathit{Id}_{H({\mathbb {D}})}, $$

where $\mathit{Id}_{X}$ denotes the identity operator on space *X*.

It is also easy to see that

6 $$ D^{j}I_{k}(f) (0)=0,\quad\mbox{for } j=\overline {0,k-1}, $$

where we regard that $D^{0}$ is the identity operator.

Beside this, by using the Newton-Leibnitz-type formula for holomorphic functions *k* times, we have

7 $$\begin{aligned}[b] I_{k}D^{k}(f) (z)&= \int_{0}^{z} \int_{0}^{\zeta_{k}}\cdots \int_{0}^{\zeta _{2}}f^{(k)}(\zeta_{1})\,d \zeta_{1} \,d\zeta_{2}\cdots d\zeta_{k} \\ &=f(z)-\sum_{j=0}^{k-1}\frac{f^{(j)}(0)}{j!}z^{j}, \end{aligned}$$

where $k\in {\mathbb {N}}$ and $f\in H({\mathbb {D}})$, from which it follows that

8 $$ I_{k}D^{k}f=f, $$

for every $f\in H({\mathbb {D}})/{\mathbb {P}}_{k-1}$, that is, $I_{k}D^{k}$ is the identity operator on $H({\mathbb {D}})/{\mathbb {P}}_{k-1}$, and consequently on its subspaces, such as are ${\mathcal {W}}^{(m)}_{\mu}/{\mathbb {P}}_{k-1}$, where $m\ge k$.

Let $\varphi \in S({\mathbb {D}})$. Then by $C_{\varphi }$ we denote the composition operator on $H({\mathbb {D}})$, which is defined by $C_{\varphi }(f)(z)=f(\varphi (z))$.

Let $u\in H({\mathbb {D}})$. Then by $M_{g}$ is denoted the multiplication operator on $H({\mathbb {D}})$, which is defined by $M_{g}(f)(z)=g(z)f(z)$.

The product of operators $C_{\varphi }$ and $M_{g}$, that is,

$$(M_{g}\circ C_{\varphi }) (f) (z)=g(z)f\bigl(\varphi (z)\bigr), $$

is called the weighted composition operator and is denoted by $gC_{\varphi }$.

These three operators have been considerably studied on various spaces of holomorphic functions (see, for example, [1, 2, 6, 11, 12] and the references therein).

Let $\varphi \in S({\mathbb {D}})$, $g\in H({\mathbb {D}})$ and $k\in {\mathbb {N}}$. We define an operator on $H({\mathbb {D}})$ as follows:

9 $$ C_{\varphi ,k}^{g}(f) (z):= \int_{0}^{z} \int_{0}^{\zeta_{k}}\cdots \int_{0}^{\zeta_{2}}f^{(k)}\bigl(\varphi ( \zeta_{1})\bigr)g(\zeta_{1})\,d\zeta_{1} \,d\zeta _{2}\cdots d\zeta_{k}, $$

for $f\in H({\mathbb {D}})$. For $k=1$ is obtained the generalized composition operator in [9], which was later studied or generalized, for example, in [10, 13–17]. For some related operators; see, also [18–28] and the references therein.

Note that from (9) it immediately follows that

10 $$ D^{j}C_{\varphi ,k}^{g}(f) (0)=0,\quad\mbox{for } j=\overline {0,k-1}. $$

Motivated by [9, 29, 30] here we calculate the essential norm of operator (9) between two *k*th weighted-type spaces. For some related results see also [6, 31].

## Main results

In this section we prove the main results in this paper.

### Theorem 1

*Assume that* *μ* *and* *ν* *are weights*, $k,m\in {\mathbb {N}}_{0}$, *and that the operator* $L:{\mathcal {W}}^{(k)}_{\mu}\to{\mathcal {W}}^{(m)}_{\nu}$ *is bounded*. *Then*

11 $$ \|L\|_{e, {\mathcal {W}}^{(k)}_{\mu}\to{\mathcal {W}}^{(m)}_{\nu}}=\|L\|_{e, {\mathcal {W}}^{(k)}_{\mu}/{\mathbb {P}}_{k-1}\to {\mathcal {W}}^{(m)}_{\nu}}. $$

### Proof

If $k=0$, then we regard that $\mathcal {W}^{(0)}_{\mu}/{\mathbb {P}}_{-1}=\mathcal {W}^{(0)}_{\mu}$, so that (11) obviously holds. Now assume that $k \in {\mathbb {N}}$. For each compact operator $K:{\mathcal {W}}^{(k)}_{\mu}\to {\mathcal {W}}^{(m)}_{\nu}$, its restriction on ${\mathcal {W}}^{(k)}_{\mu}/{\mathbb {P}}_{k-1}$, that is, $K:{\mathcal {W}}^{(k)}_{\mu}/{\mathbb {P}}_{k-1}\to {\mathcal {W}}^{(m)}_{\nu}$, is also a compact operator, from which along with the definition of the essential norm of an operator, it easily follows that

12 $$ \|L\|_{e, {\mathcal {W}}^{(k)}_{\mu}/{\mathbb {P}}_{k-1}\to {\mathcal {W}}^{(m)}_{\nu}}\le\|L\|_{e, {\mathcal {W}}^{(k)}_{\mu}\to {\mathcal {W}}^{(m)}_{\nu}}. $$

Let $K:{\mathcal {W}}^{(k)}_{\mu}/{\mathbb {P}}_{k-1}\to{\mathcal {W}}^{(m)}_{\nu}$ be a compact operator and $f\in{\mathcal {W}}^{(k)}_{\mu}$. Then by

$$\widetilde{K} (f) (z)=K \Biggl(f-\sum_{j=0}^{k-1}a_{j}z^{j} \Biggr), $$

where $a_{j}=f^{(j)}(0)/j!$, $j=\overline {0,k-1}$, is defined an extension of operator *K* on the whole space ${\mathcal {W}}^{(k)}_{\mu}$, that is, $\widetilde{K}:{\mathcal {W}}^{(k)}_{\mu}\to{\mathcal {W}}^{(m)}_{\nu}$, which is obviously a compact operator. Denote the set of such obtained operators *K̃* by $\widetilde{\mathcal {K}}$.

Let $L_{1}:{\mathcal {W}}^{(k)}_{\mu}\to{\mathcal {W}}^{(m)}_{\mu}$ be a bounded operator, then the operator

$$\widetilde{L}_{1}(f):=L_{1} \Biggl(\sum_{j=0}^{k-1}a_{j}z^{j} \Biggr)=\sum_{j=0}^{k-1}a_{j}L_{1} \bigl(z^{j}\bigr), $$

where, as above, $a_{j} = f^{(j)}(0)/j!$, $j=\overline{0,k-1}$, maps ${\mathcal {W}}^{(k)}_{\mu}$ into ${\mathcal {W}}^{(m)}_{\mu}$, and is compact, since its image is a finite-dimensional space.

We have

13 $$\begin{aligned}[b] &\|L\|_{e, {\mathcal {W}}^{(k)}_{\mu}\to{\mathcal {W}}^{(m)}_{\nu}}\\ &\quad=\inf_{K_{1}\in{\mathcal {K}}({\mathcal {W}}^{(k)}_{\mu},{\mathcal {W}}^{(m)}_{\nu})}\|L-K_{1} \|_{{\mathcal {W}}^{(k)}_{\mu}\to {\mathcal {W}}^{(m)}_{\nu}} \\ &\quad\le\inf_{\widetilde{K}\in{\mathcal {K}}({\mathcal {W}}^{(k)}_{\mu},{\mathcal {W}}^{(m)}_{\nu})\cap\widetilde{\mathcal {K}}}\|L-\widetilde{K}-\widetilde{L}\|_{{\mathcal {W}}^{(k)}_{\mu}\to{\mathcal {W}}^{(m)}_{\nu}} \\ &\quad=\inf_{\widetilde{K}\in{\mathcal {K}}({\mathcal {W}}^{(k)}_{\mu},{\mathcal {W}}^{(m)}_{\nu})\cap\widetilde{\mathcal {K}}} \sup_{\|f\|_{{\mathcal {W}}^{(k)}_{\mu}}\le1}\big\| L(f)-\widetilde{K}(f)-\widetilde{L}(f)\big\| _{{\mathcal {W}}^{(m)}_{\nu}} \\ &\quad=\inf_{K\in{\mathcal {K}}({\mathcal {W}}^{(k)}_{\mu}/{\mathbb {P}}_{k-1},{\mathcal {W}}^{(m)}_{\nu})}\sup_{\|f\|_{{\mathcal {W}}^{(k)}_{\mu}}\le1}\Bigg\| L(f)-K \Biggl(f- \sum_{j=0}^{k-1}a_{j}z^{j} \Biggr)-L \Biggl(\sum_{j=0}^{k-1}a_{j}z^{j} \Biggr) \Bigg\| _{{\mathcal {W}}^{(m)}_{\nu}} \\ &\quad=\inf_{K\in{\mathcal {K}}({\mathcal {W}}^{(k)}_{\mu}/{\mathbb {P}}_{k-1},{\mathcal {W}}^{(m)}_{\nu})}\sup_{\|f\|_{{\mathcal {W}}^{(k)}_{\mu}}\le1} \Bigg\| (L-K) \Biggl(f- \sum_{j=0}^{k-1}a_{j}z^{j} \Biggr) \Bigg\| _{{\mathcal {W}}^{(m)}_{\nu}} \\ &\quad\le\inf_{K\in{\mathcal {K}}({\mathcal {W}}^{(k)}_{\mu}/{\mathbb {P}}_{k-1},{\mathcal {W}}^{(m)}_{\nu})}\sup_{\{g\in{\mathcal {W}}^{(k)}_{\mu}/{\mathbb {P}}_{k-1}:\|g\|_{{\mathcal {W}}^{(k)}_{\mu}}\le1\}}\big\| (L-K) (g)\big\| _{{\mathcal {W}}^{(m)}_{\nu}} \\ &\quad=\inf_{K\in{\mathcal {K}}({\mathcal {W}}^{(k)}_{\mu}/{\mathbb {P}}_{k-1},{\mathcal {W}}^{(m)}_{\nu})}\|L-K\|_{{\mathcal {W}}^{(k)}_{\mu}/{\mathbb {P}}_{k-1}\to{\mathcal {W}}^{(m)}_{\nu}}=\|L\|_{e, {\mathcal {W}}^{(k)}_{\mu}/{\mathbb {P}}_{k-1}\to {\mathcal {W}}^{(m)}_{\nu}}.\end{aligned} $$

From (12) and (13), equality (11) follows. □

### Theorem 2

*Assume that* *μ* *and* *ν* *are weights*, $k,l,m\in {\mathbb {N}}$, $m\ge k$, *and that the operator* $C_{\varphi ,k}^{g}:{\mathcal {W}}^{(m+l-1)}_{\mu}\to{\mathcal {W}}^{(m)}_{\nu}$ *is bounded*. *Then*

14 $$ \big\| C_{\varphi ,k}^{g}\big\| _{e, {\mathcal {W}}^{(m+l-1)}_{\mu}\to{\mathcal {W}}^{(m)}_{\nu}}=\|gC_{\varphi } \|_{e, {\mathcal {W}}^{(m+l-k-1)}_{\mu}\to {\mathcal {W}}^{(m-k)}_{\nu}}. $$

### Proof

First we prove the following inequality:

15 $$ \big\| C_{\varphi ,k}^{g}\big\| _{e, {\mathcal {W}}^{(m+l-1)}_{\mu}\to{\mathcal {W}}^{(m)}_{\nu}}\le\|gC_{\varphi } \|_{e, {\mathcal {W}}^{(m+l-k-1)}_{\mu}\to {\mathcal {W}}^{(m-k)}_{\nu}}. $$

We show that

16 $$ \big\| C_{\varphi ,k}^{g}\big\| _{e, {\mathcal {W}}^{(m+l-1)}_{\mu}/{\mathbb {P}}_{m+l-2}\to {\mathcal {W}}^{(m)}_{\nu}}\le\|gC_{\varphi } \|_{e, {\mathcal {W}}^{(m+l-k-1)}_{\mu}/{\mathbb {P}}_{m+l-k-2}\to{\mathcal {W}}^{(m-k)}_{\nu}}, $$

which by Theorem 1 is equivalent to (15) (recall that when $m=k$ and $l=1$, we naturally regard that ${\mathcal {W}}^{(m+l-k-1)}_{\mu}/{\mathbb {P}}_{m+l-k-2}={\mathcal {W}}^{(0)}_{\mu}$).

Assume that $f\in{\mathcal {W}}^{(m+l-k-1)}_{\mu}/{\mathbb {P}}_{m+l-k-2}$. Then, since

17 $$ \|I_{k}f\|_{{\mathcal {W}}^{(m+l-1)}_{\mu}}=\|f\|_{{\mathcal {W}}^{(m+l-k-1)}_{\mu}}< +\infty $$

and

18 $$ D^{j}(I_{k}f) (0)=0,\quad\mbox{for } j=\overline {0,m+l-2}, $$

it follows that $I_{k}f\in{\mathcal {W}}^{(m+l-1)}_{\mu}/{\mathbb {P}}_{m+l-2}$, that is, operator $I_{k}$ maps the space ${\mathcal {W}}^{(m+l-k-1)}_{\mu}/{\mathbb {P}}_{m+l-k-2}$ into ${\mathcal {W}}^{(m+l-1)}_{\mu}/{\mathbb {P}}_{m+l-2}$. Further, it is clear that $C_{\varphi ,k}^{g}:{\mathcal {W}}^{(m+l-1)}_{\mu}/{\mathbb {P}}_{m+l-2}\to{\mathcal {W}}^{(m)}_{\nu}$ is bounded, and since for every $h\in{\mathcal {W}}^{(m)}_{\nu}$,

19 $$ b_{{\mathcal {W}}^{(m-k)}_{\nu}}\bigl(D^{k} h\bigr)=b_{{\mathcal {W}}^{(m)}_{\nu}}(h)< \infty, $$

we see that $D^{k}$ maps ${\mathcal {W}}^{(m)}_{\nu}$ into ${\mathcal {W}}^{(m-k)}_{\nu}$. Moreover, we have

20 $$ \big\| D^{k} h\big\| _{{\mathcal {W}}^{(m-k)}_{\nu}}\le\|h\| _{{\mathcal {W}}^{(m)}_{\nu}}, $$

where the strict inequality can occur here.

Hence, we see that the operator

21 $$ D^{k}C_{\varphi ,k}^{g}I_{k}=gC_{\varphi } $$

maps the space ${\mathcal {W}}^{(m+l-k-1)}_{\mu}/{\mathbb {P}}_{m+l-k-2}$ into ${\mathcal {W}}^{(m-k)}_{\nu}$, and from (17), (20) and the boundedness of the operator $C_{\varphi ,k}^{g}:{\mathcal {W}}^{(m+l-1)}_{\mu}/{\mathbb {P}}_{m+l-2}\to{\mathcal {W}}^{(m)}_{\nu}$, it follows that the operator $gC_{\varphi }:{\mathcal {W}}^{(m+l-k-1)}_{\mu}/{\mathbb {P}}_{m+l-k-2}\to{\mathcal {W}}^{(m-k)}_{\nu}$ is also bounded.

Due to (8) and (10), we have

22 $$ I_{k}D^{k}C_{\varphi ,k}^{g}I_{k}D^{k}f=C_{\varphi ,k}^{g}f, $$

for every $f\in{\mathcal {W}}^{(m+l-1)}_{\mu}/{\mathbb {P}}_{m+l-2}$. Indeed, since $m\ge k$ and $l\in {\mathbb {N}}$ we have $m+l-1\ge k$, so by (8) we have $I_{k}D^{k}f=f$, and further from (10) we get $C_{\varphi ,k}^{g}f\in{\mathcal {W}}^{(m)}_{\mu}/{\mathbb {P}}_{k-1}$. By another application of (8) is obtained (22).

Let $K:{\mathcal {W}}^{(m+l-k-1)}_{\mu}/{\mathbb {P}}_{m+l-k-2}\to{\mathcal {W}}^{(m-k)}_{\nu}$ be a compact operator and

23 $$ \widetilde{K}:=I_{k}KD^{k}. $$

Then the operator maps the space ${\mathcal {W}}^{(m+l-1)}_{\mu}/{\mathbb {P}}_{m+l-2}$ into ${\mathcal {W}}^{(m)}_{\nu}$ and is compact, since the space of compact operators is a both sided ideal into the space of bounded linear operators.

Hence, by using (21)-(23) and some simple estimates, we have

24 $$\begin{aligned}[b] \big\| C_{\varphi ,k}^{g}f-\widetilde{K}f\big\| _{{\mathcal {W}}^{(m)}_{\nu}} &= \big\| I_{k}D^{k}C_{\varphi ,k}^{g}I_{k}D^{k}f-I_{k}KD^{k}f \big\| _{{\mathcal {W}}^{(m)}_{\nu}} \\ &=\big\| I_{k}\bigl(D^{k}C_{\varphi ,k}^{g}I_{k}-K \bigr)D^{k}f\big\| _{{\mathcal {W}}^{(m)}_{\nu}} \\ &=\big\| (gC_{\varphi }-K)D^{k}f\big\| _{{\mathcal {W}}^{(m-k)}_{\nu}} \\ &\le\|gC_{\varphi }-K\|_{{\mathcal {W}}^{(m+l-k-1)}_{\mu}/{\mathbb {P}}_{m+l-k-2}\to {\mathcal {W}}^{(m-k)}_{\nu}}\big\| D^{k}f\big\| _{{\mathcal {W}}^{(m+l-k-1)}_{\mu}} \\ &\le\|gC_{\varphi }-K\|_{{\mathcal {W}}^{(m+l-k-1)}_{\mu}/{\mathbb {P}}_{m+l-k-2}\to {\mathcal {W}}^{(m-k)}_{\nu}}\|f\|_{{\mathcal {W}}^{(m+l-1)}_{\mu}}.\end{aligned} $$

By taking the supremum in (24) over the unit ball in ${\mathcal {W}}^{(m+l-1)}_{\mu}/{\mathbb {P}}_{m+l-2}$, and then taking the infimum in such obtained inequality over the set of all compact operators $K:{\mathcal {W}}^{(m+l-k-1)}_{\mu}/{\mathbb {P}}_{m+l-k-2}\to{\mathcal {W}}^{(m-k)}_{\nu}$, we get (16).

Now we prove the following inequality:

25 $$ \|gC_{\varphi }\|_{e, {\mathcal {W}}^{(m+l-k-1)}_{\mu}\to{\mathcal {W}}^{(m-k)}_{\nu}}\le\big\| C_{\varphi ,k}^{g} \big\| _{e, {\mathcal {W}}^{(m+l-1)}_{\mu}\to {\mathcal {W}}^{(m)}_{\nu}}. $$

To do this first note that since (17) holds for every $f\in {\mathcal {W}}^{(m+l-k-1)}_{\mu}$, the operator $I_{k}:{\mathcal {W}}^{(m+l-k-1)}_{\mu}\to{\mathcal {W}}^{(m+l-1)}_{\mu}$ is bounded. From this, since $C_{\varphi ,k}^{g}:{\mathcal {W}}^{(m+l-1)}_{\mu}\to{\mathcal {W}}^{(m)}_{\nu}$ is bounded by the assumption, and $D^{k}:{\mathcal {W}}^{(m)}_{\mu}\to{\mathcal {W}}^{(m-k)}_{\nu}$ is also bounded due to the inequality in (20), we see that the operator $D^{k}C_{\varphi ,k}^{g}I_{k}=gC_{\varphi }:{\mathcal {W}}^{(m+l-k-1)}_{\mu}\to{\mathcal {W}}^{(m-k)}_{\nu}$ is bounded.

Note also that

26 $$ C_{\varphi ,k}^{g}=I_{k}gC_{\varphi }D^{k}, $$

where $D^{k}:{\mathcal {W}}^{(m+l-1)}_{\mu}\to{\mathcal {W}}^{(m+l-k-1)}_{\mu}$ and $I_{k}:{\mathcal {W}}^{(m-k)}_{\nu}\to{\mathcal {W}}^{(m)}_{\nu}$.

Let $K:{\mathcal {W}}^{(m+l-1)}_{\mu}\to{\mathcal {W}}^{(m)}_{\nu}$ be a compact operator. Then the operator

$$D^{k}KI_{k}:{\mathcal {W}}^{(m+l-k-1)}_{\mu}\to{ \mathcal {W}}^{(m-k)}_{\nu}$$

is compact too.

Using this facts and (5), it follows that

27 $$\begin{aligned}[b] \big\| gC_{\varphi }f-D^{k}KI_{k}f\big\| _{{\mathcal {W}}^{(m-k)}_{\nu}} &= \big\| D^{k}I_{k}gC_{\varphi }D^{k}I_{k}f-D^{k}KI_{k}f \big\| _{{\mathcal {W}}^{(m-k)}_{\nu}} \\ &=\big\| D^{k}\bigl(C_{\varphi ,k}^{g}-K\bigr)I_{k}f \big\| _{{\mathcal {W}}^{(m-k)}_{\nu}} \\ &\le\big\| \bigl(C_{\varphi ,k}^{g}-K\bigr)I_{k}f \big\| _{{\mathcal {W}}^{(m)}_{\nu}} \\ &\le\big\| C_{\varphi ,k}^{g}-K\big\| _{{\mathcal {W}}^{(m+l-1)}_{\mu}\to{\mathcal {W}}^{(m)}_{\nu}}\|I_{k}f \|_{{\mathcal {W}}^{(m+l-1)}_{\mu}} \\ &\le\big\| C_{\varphi ,k}^{g}-K\big\| _{{\mathcal {W}}^{(m+l-1)}_{\mu}\to{\mathcal {W}}^{(m)}_{\nu}}\|f\|_{{\mathcal {W}}^{(m+l-k-1)}_{\mu}}.\end{aligned} $$

By taking the supremum in (27) over the unit ball in ${\mathcal {W}}^{(m+l-k-1)}_{\mu}$, and then taking the infimum in such obtained inequality over the set of all compact operators $K:{\mathcal {W}}^{(m+l-1)}_{\mu}\to{\mathcal {W}}^{(m)}_{\nu}$, the inequality (25) is obtained. From (15) and (25) equality (14) follows. □

Before we formulate our next results, we want to say that their proofs are related to the one of Theorem 2, but we will give all the differences for the completeness.

### Theorem 3

*Assume that* *μ* *and* *ν* *are weights*, $k,l,m\in {\mathbb {N}}$, $m\ge k$, *and that the operator* $C_{\varphi ,k}^{g}:{\mathcal {W}}^{(m)}_{\mu}\to{\mathcal {W}}^{(m+l-1)}_{\nu}$ *is bounded*. *Then*

28 $$ \big\| C_{\varphi ,k}^{g}\big\| _{e, {\mathcal {W}}^{(m)}_{\mu}\to{\mathcal {W}}^{(m+l-1)}_{\nu}}=\|gC_{\varphi } \|_{e, {\mathcal {W}}^{(m-k)}_{\mu}\to {\mathcal {W}}^{(m+l-k-1)}_{\nu}}. $$

### Proof

First we prove

29 $$ \big\| C_{\varphi ,k}^{g}\big\| _{e, {\mathcal {W}}^{(m)}_{\mu}\to{\mathcal {W}}^{(m+l-1)}_{\nu}}\le\|gC_{\varphi } \|_{e, {\mathcal {W}}^{(m-k)}_{\mu}\to {\mathcal {W}}^{(m+l-k-1)}_{\nu}}. $$

We show that

30 $$ \big\| C_{\varphi ,k}^{g}\big\| _{e, {\mathcal {W}}^{(m)}_{\mu}/{\mathbb {P}}_{m-1}\to{\mathcal {W}}^{(m+l-1)}_{\nu}}\le\|gC_{\varphi } \|_{e, {\mathcal {W}}^{(m-k)}_{\mu}/{\mathbb {P}}_{m-k-1}\to{\mathcal {W}}^{(m+l-k-1)}_{\nu}}, $$

which by Theorem 1 is equivalent to (29).

Assume that $f\in{\mathcal {W}}^{(m-k)}_{\mu}/{\mathbb {P}}_{m-k-1}$. Then, since

31 $$ \|I_{k}f\|_{{\mathcal {W}}^{(m)}_{\mu}}=\|f\|_{{\mathcal {W}}^{(m-k)}_{\mu}}< \infty $$

and

32 $$ D^{j}(I_{k}f) (0)=0,\quad\mbox{for } j=\overline {0,m-1}, $$

it follows that $I_{k}f\in{\mathcal {W}}^{(m)}_{\mu}/{\mathbb {P}}_{m-1}$, that is, operator $I_{k}$ maps space ${\mathcal {W}}^{(m-k)}_{\mu}/{\mathbb {P}}_{m-k-1}$ into ${\mathcal {W}}^{(m)}_{\mu}/{\mathbb {P}}_{m-1}$. Further, it is clear that $C_{\varphi ,k}^{g}:{\mathcal {W}}^{(m)}_{\mu}/{\mathbb {P}}_{m-1}\to{\mathcal {W}}^{(m+l-1)}_{\nu}$ is bounded, and since for every $h\in{\mathcal {W}}^{(m+l-1)}_{\nu}$,

33 $$ b_{{\mathcal {W}}^{(m+l-k-1)}_{\mu}}\bigl(D^{k} h\bigr)=b_{{\mathcal {W}}^{(m+l-1)}_{\mu}}(h)< + \infty $$

we see that $D^{k}:{\mathcal {W}}^{(m+l-1)}_{\nu}\to{\mathcal {W}}^{(m+l-k-1)}_{\nu}$. Moreover, we have

34 $$ \big\| D^{k} h\big\| _{{\mathcal {W}}^{(m+l-k-1)}_{\mu}}\le\|h\| _{{\mathcal {W}}^{(m+l-1)}_{\mu}}. $$

Hence, we see that the operator (21) maps ${\mathcal {W}}^{(m-k)}_{\mu}/{\mathbb {P}}_{m-k-1}$ to ${\mathcal {W}}^{(m+l-k-1)}$, and from (31), (34) and the boundedness of $C_{\varphi ,k}^{g}:{\mathcal {W}}^{(m)}_{\mu}/{\mathbb {P}}_{m-1}\to {\mathcal {W}}^{(m+l-1)}_{\nu}$ it follows that the operator $gC_{\varphi }:{\mathcal {W}}^{(m-k)}_{\mu}/{\mathbb {P}}_{m-k-1}\to{\mathcal {W}}^{(m+l-k-1)}$ is also bounded. Beside this, since $m\ge k$, we see that (22) holds for every $f\in{\mathcal {W}}^{(m)}_{\mu}/{\mathbb {P}}_{m-1}$.

Let $K:{\mathcal {W}}^{(m-k)}_{\mu}/{\mathbb {P}}_{m-k-1}\to{\mathcal {W}}^{(m+l-k-1)}_{\nu}$ be a compact operator. Then the operator

$$\widetilde{K}:=I_{k}KD^{k}:{\mathcal {W}}^{(m)}_{\mu}/ {\mathbb {P}}_{m-1}\to{\mathcal {W}}^{(m+l-1)}_{\nu}$$

is compact.

From this, (21) and (22), we have

35 $$\begin{aligned}[b] \big\| C_{\varphi ,k}^{g}f-\widetilde{K}f\big\| _{{\mathcal {W}}^{(m+l-1)}_{\nu}} &= \big\| I_{k}D^{k}C_{\varphi ,k}^{g}I_{k}D^{k}f-I_{k}KD^{k}f \big\| _{{\mathcal {W}}^{(m+l-1)}_{\nu}} \\ &=\big\| I_{k}\bigl(D^{k}C_{\varphi ,k}^{g}I_{k}-K \bigr)D^{k}f\big\| _{{\mathcal {W}}^{(m+l-1)}_{\nu}} \\ &=\big\| (gC_{\varphi }-K)D^{k}f\big\| _{{\mathcal {W}}^{(m+l-k-1)}_{\nu}} \\ &\le\|gC_{\varphi }-K\|_{{\mathcal {W}}^{(m-k)}_{\mu}/{\mathbb {P}}_{m-k-1}\to {\mathcal {W}}^{(m+l-k-1)}}\big\| D^{k}f\big\| _{{\mathcal {W}}^{(m-k)}_{\mu}/{\mathbb {P}}_{m-k-1}} \\ &\le\|gC_{\varphi }-K\|_{{\mathcal {W}}^{(m-k)}_{\mu}/{\mathbb {P}}_{m-k-1}\to {\mathcal {W}}^{(m+l-k-1)}}\|f\|_{{\mathcal {W}}^{(m)}_{\mu}}.\end{aligned} $$

By taking the supremum in (35) over the unit ball in ${\mathcal {W}}^{(m)}_{\mu}/{\mathbb {P}}_{m-1}$, and then taking the infimum in such obtained inequality over the set of all compact operators $K:{\mathcal {W}}^{(m-k)}_{\mu}/{\mathbb {P}}_{m-k-1}\to{\mathcal {W}}^{(m+l-k-1)}_{\nu}$, we get (30).

Now we prove that

36 $$ \|gC_{\varphi }\|_{e, {\mathcal {W}}^{(m-k)}_{\mu}\to{\mathcal {W}}^{(m+l-k-1)}_{\nu}}\le\big\| C_{\varphi ,k}^{g} \big\| _{e, {\mathcal {W}}^{(m)}_{\mu}\to {\mathcal {W}}^{(m+l-1)}_{\nu}}. $$

Since (31) holds for every $f\in{\mathcal {W}}^{(m-k)}_{\mu}$, we see that the operator $I_{k}:{\mathcal {W}}^{(m-k)}_{\mu}\to{\mathcal {W}}^{(m)}_{\mu}$ is bounded. From this, since $C_{\varphi ,k}^{g}:{\mathcal {W}}^{(m)}_{\mu}\to{\mathcal {W}}^{(m+l-1)}_{\nu}$ is bounded by the assumption, and $D^{k}:{\mathcal {W}}^{(m+l-1)}_{\nu}\to{\mathcal {W}}^{(m+l-k-1)}_{\nu}$ is also bounded due to the inequality in (34), we see that the operator $D^{k}C_{\varphi ,k}^{g}I_{k}=gC_{\varphi }:{\mathcal {W}}^{(m-k)}_{\mu}\to{\mathcal {W}}^{(m+l-k-1)}_{\nu}$ is bounded. Note also that (26) holds, where $D^{k}:{\mathcal {W}}^{(m)}_{\mu}\to{\mathcal {W}}^{(m-k)}_{\nu}$ and $I_{k}:{\mathcal {W}}^{(m+l-k-1)}_{\mu}\to{\mathcal {W}}^{(m+l-1)}_{\nu}$.

Let $K:{\mathcal {W}}^{(m)}_{\mu}\to{\mathcal {W}}^{(m+l-1)}_{\nu}$ be a compact operator. Then the operator

$$D^{k}KI_{k}:{\mathcal {W}}^{(m-k)}_{\mu}\to{ \mathcal {W}}^{(m+l-k-1)}_{\nu}$$

is compact.

Using this fact along with (5) and (26), we have

37 $$\begin{aligned}[b] \big\| gC_{\varphi }f-D^{k}KI_{k}f\big\| _{{\mathcal {W}}^{(m+l-k-1)}_{\nu}} &= \big\| D^{k}I_{k}gC_{\varphi }D^{k}I_{k}f-D^{k}KI_{k}f \big\| _{{\mathcal {W}}^{(m+l-k-1)}_{\nu}} \\ &=\big\| D^{k}\bigl(C_{\varphi ,k}^{g}-K\bigr)I_{k}f \big\| _{{\mathcal {W}}^{(m+l-k-1)}_{\nu}} \\ &\le\big\| \bigl(C_{\varphi ,k}^{g}-K\bigr)I_{k}f \big\| _{{\mathcal {W}}^{(m+l-1)}_{\nu}} \\ &\le\big\| C_{\varphi ,k}^{g}-K\big\| _{{\mathcal {W}}^{(m)}_{\mu}\to{\mathcal {W}}^{(m+l-1)}_{\nu}}\|I_{k}f \|_{{\mathcal {W}}^{(m)}_{\mu}} \\ &\le\big\| C_{\varphi ,k}^{g}-K\big\| _{{\mathcal {W}}^{(m)}_{\mu}\to{\mathcal {W}}^{(m+l-1)}_{\nu}}\|f\|_{{\mathcal {W}}^{(m-k)}_{\mu}}.\end{aligned} $$

By taking the supremum in (37) over the unit ball in ${\mathcal {W}}^{(m-k)}_{\mu}$, and then taking the infimum in such obtained inequality over the set of all compact operators $K:{\mathcal {W}}^{(m)}_{\mu}\to{\mathcal {W}}^{(m+l-1)}_{\nu}$, we get (36). From (29) and (36) is directly obtained (28), as desired. □

### Theorem 4

*Assume that* *μ* *and* *ν* *are weights*, $k,l,m\in {\mathbb {N}}$, $m\ge k$, *and that the operator* $C_{\varphi ,k}^{g}:{\mathcal {W}}^{(m+l-1)}_{\mu}/{\mathbb {P}}_{m+l-2}\to{\mathcal {W}}^{(m)}_{\nu}/{\mathbb {P}}_{m-1}$ *is bounded*. *Then*

38 $$ \big\| C_{\varphi ,k}^{g}\big\| _{e, {\mathcal {W}}^{(m+l-1)}_{\mu}/ {\mathbb {P}}_{m+l-2}\to{\mathcal {W}}^{(m)}_{\nu}/ {\mathbb {P}}_{m-1}} = \|gC_{\varphi } \|_{e, {\mathcal {W}}^{(m+l-k-1)}_{\mu}/ {\mathbb {P}}_{m+l-k-2}\to{\mathcal {W}}^{(m-k)}_{\nu}/ {\mathbb {P}}_{m-k-1}} . $$

### Proof

First we prove the following inequality:

39 $$ \big\| C_{\varphi ,k}^{g}\big\| _{e, {\mathcal {W}}^{(m+l-1)}_{\mu}/ {\mathbb {P}}_{m+l-2}\to{\mathcal {W}}^{(m)}_{\nu}/ {\mathbb {P}}_{m-1}} \le\| gC_{\varphi } \|_{e, {\mathcal {W}}^{(m+l-k-1)}_{\mu}/ {\mathbb {P}}_{m+l-k-2}\to {\mathcal {W}}^{(m-k)}_{\nu}/ {\mathbb {P}}_{m-k-1}}. $$

Assume that $f\in{\mathcal {W}}^{(m+l-k-1)}_{\mu}/{\mathbb {P}}_{m+l-k-2}$. Then, since (17) and (18) hold, we see that the operator $I_{k}$ maps the space ${\mathcal {W}}^{(m+l-k-1)}_{\mu}/{\mathbb {P}}_{m+l-k-2}$ into ${\mathcal {W}}^{(m+l-1)}_{\mu}/{\mathbb {P}}_{m+l-2}$. Further, by the assumption $C_{\varphi ,k}^{g}:{\mathcal {W}}^{(m+l-1)}_{\mu}/{\mathbb {P}}_{m+l-2}\to{\mathcal {W}}^{(m)}_{\nu}/{\mathbb {P}}_{m-1}$ is bounded, and since for every $h\in{\mathcal {W}}^{(m)}_{\nu}/{\mathbb {P}}_{m-1}$, (19) holds and

40 $$ D^{j}\bigl(D^{k} h\bigr) (0)=0,\quad j=\overline {0,m-k-1}, $$

we have $D^{k}:{\mathcal {W}}^{(m)}_{\nu}/{\mathbb {P}}_{m-1}\to{\mathcal {W}}^{(m-k)}_{\nu}/{\mathbb {P}}_{m-k-1}$. Moreover, we see that (20) holds.

Hence, we see that the operator (21) maps the space ${\mathcal {W}}^{(m+l-k-1)}_{\mu}/{\mathbb {P}}_{m+l-k-2}$ into ${\mathcal {W}}^{(m-k)}_{\nu}/{\mathbb {P}}_{m-k-1}$, and from (17), (20) and the boundedness of the operator $C_{\varphi ,k}^{g}: {\mathcal {W}}^{(m+l-1)}_{\mu}/{\mathbb {P}}_{m+l-2}\to{\mathcal {W}}^{(m)}_{\nu}/{\mathbb {P}}_{m-1}$, it follows that $gC_{\varphi }:{\mathcal {W}}^{(m+l-k-1)}_{\mu}/{\mathbb {P}}_{m+l-k-2}\to{\mathcal {W}}^{(m-k)}_{\nu}/{\mathbb {P}}_{m-k-1}$ is also bounded. Since $m+l-1\ge k$, we see that (22) holds for every $f\in{\mathcal {W}}^{(m+l-1)}_{\mu}/{\mathbb {P}}_{m+l-2}$.

Let $K:{\mathcal {W}}^{(m+l-k-1)}_{\mu}/{\mathbb {P}}_{m+l-k-2}\to{\mathcal {W}}^{(m-k)}_{\nu}/{\mathbb {P}}_{m-k-1}$ be a compact operator and *K̃* be defined as in (23), where $D^{k}$ maps the space ${\mathcal {W}}^{(m+l-1)}_{\mu}/{\mathbb {P}}_{m+l-2}$ into ${\mathcal {W}}^{(m+l-k-1)}_{\mu}/{\mathbb {P}}_{m+l-k-2}$, and $I_{k}$ maps the space ${\mathcal {W}}^{(m-k)}_{\nu}/{\mathbb {P}}_{m-k-1}$ into ${\mathcal {W}}^{(m)}_{\nu}/{\mathbb {P}}_{m-1}$. Then, *K̃* maps the space ${\mathcal {W}}^{(m+l-1)}_{\mu}/{\mathbb {P}}_{m+l-2}$ into ${\mathcal {W}}^{(m)}_{\nu}/{\mathbb {P}}_{m-1}$ and is compact.

From this and (21), similar to (24), is obtained

41 $$ \big\| C_{\varphi ,k}^{g}f-\widetilde{K}f\big\| _{{\mathcal {W}}^{(m)}_{\nu}} \le \|gC_{\varphi }-K\|_{{\mathcal {W}}^{(m+l-k-1)}_{\mu}/{\mathbb {P}}_{m+l-k-2}\to {\mathcal {W}}^{(m-k)}_{\nu}/{\mathbb {P}}_{m-k-1}}\|f\|_{{\mathcal {W}}^{(m+l-1)}_{\mu}}. $$

By taking the supremum in (41) over the unit ball in ${\mathcal {W}}^{(m+l-1)}_{\mu}/{\mathbb {P}}_{m+l-2}$, and then taking the infimum in such obtained inequality over the set of all compact operators $K:{\mathcal {W}}^{(m+l-k-1)}_{\mu}/{\mathbb {P}}_{m+l-k-2}\to{\mathcal {W}}^{(m-k)}_{\nu}/{\mathbb {P}}_{m-k-1}$, we get (39).

Now we prove that

42 $$ \|gC_{\varphi }\|_{e, {\mathcal {W}}^{(m+l-k-1)}_{\mu}/ {\mathbb {P}}_{m+l-k-2}\to{\mathcal {W}}^{(m-k)}_{\nu}/ {\mathbb {P}}_{m-k-1}} \le \big\| C_{\varphi ,k}^{g} \big\| _{e, {\mathcal {W}}^{(m+l-1)}_{\mu}/ {\mathbb {P}}_{m+l-2}\to {\mathcal {W}}^{(m)}_{\nu}/ {\mathbb {P}}_{m-1}}. $$

Since (17) holds for every $f\in{\mathcal {W}}^{(m+l-k-1)}_{\mu}/{\mathbb {P}}_{m+l-k-2}$, the operator $I_{k}:{\mathcal {W}}^{(m+l-k-1)}_{\mu}/ {\mathbb {P}}_{m+l-k-2}\to{\mathcal {W}}^{(m+l-1)}_{\mu}/{\mathbb {P}}_{m+l-2}$ is bounded. From this, since $C_{\varphi ,k}^{g}:{\mathcal {W}}^{(m+l-1)}_{\mu}/{\mathbb {P}}_{m+l-2}\to {\mathcal {W}}^{(m)}_{\nu}/{\mathbb {P}}_{m-1}$ is bounded, and since $D^{k}:{\mathcal {W}}^{(m)}_{\mu}/{\mathbb {P}}_{m-1}\to{\mathcal {W}}^{(m-k)}_{\nu}/{\mathbb {P}}_{m-k-1}$ is also bounded due to (20) and (40), we see that the operator $D^{k}C_{\varphi ,k}^{g}I_{k}=gC_{\varphi }:{\mathcal {W}}^{(m+l-k-1)}_{\mu}/{\mathbb {P}}_{m+l-k-2}\to{\mathcal {W}}^{(m-k)}_{\nu}/{\mathbb {P}}_{m-k-1}$ is bounded. Beside this, note that $C_{\varphi ,k}^{g}=I_{k}gC_{\varphi }D^{k}:{\mathcal {W}}^{(m+l-1)}_{\mu}/{\mathbb {P}}_{m+l-2}\to{\mathcal {W}}^{(m)}_{\nu}/{\mathbb {P}}_{m-1}$, where $D^{k}:{\mathcal {W}}^{(m+l-1)}_{\mu}/{\mathbb {P}}_{m+l-2}\to {\mathcal {W}}^{(m+l-k-1)}_{\mu}/{\mathbb {P}}_{m+l-k-2}$ and $I_{k}:{\mathcal {W}}^{(m-k)}_{\nu}/{\mathbb {P}}_{m-k-1}\to{\mathcal {W}}^{(m)}_{\nu}/{\mathbb {P}}_{m-1}$.

Let $K:{\mathcal {W}}^{(m+l-1)}_{\mu}/{\mathbb {P}}_{m+l-2}\to{\mathcal {W}}^{(m)}_{\nu}/{\mathbb {P}}_{m-1}$ be a compact operator. Then the operator

$$D^{k}KI_{k}:{\mathcal {W}}^{(m+l-k-1)}_{\mu}/ {\mathbb {P}}_{m+l-k-2}\to{\mathcal {W}}^{(m-k)}_{\nu}/ {\mathbb {P}}_{m-k-1} $$

is compact too.

Hence, as in (27) we have

43 $$ \big\| gC_{\varphi }f-D^{k}KI_{k}f\big\| _{{\mathcal {W}}^{(m-k)}_{\nu}} \le \big\| C_{\varphi ,k}^{g}-K\big\| _{{\mathcal {W}}^{(m+l-1)}_{\mu}/{\mathbb {P}}_{m+l-2}\to {\mathcal {W}}^{(m)}_{\nu}/{\mathbb {P}}_{m-1}}\|f\|_{{\mathcal {W}}^{(m+l-k-1)}_{\mu}}. $$

By taking the supremum in (43) over the unit ball in ${\mathcal {W}}^{(m+l-k-1)}_{\mu}/{\mathbb {P}}_{m+l-k-2}$, and then taking the infimum in such obtained inequality over the set of all compact operators $K:{\mathcal {W}}^{(m+l-1)}_{\mu}/{\mathbb {P}}_{m+l-2}\to{\mathcal {W}}^{(m)}_{\nu}/{\mathbb {P}}_{m-1}$, we get (42). From (39) and (42) is obtained (38). □

### Theorem 5

*Assume that* *μ* *and* *ν* *are weights*, $k,l,m\in {\mathbb {N}}$, $m\ge k$, *and that the operator* $C_{\varphi ,k}^{g}:{\mathcal {W}}^{(m)}_{\mu}/{\mathbb {P}}_{m-1}\to{\mathcal {W}}^{(m+l-1)}_{\nu}/{\mathbb {P}}_{m+l-2}$ *is bounded*. *Then*

44 $$ \big\| C_{\varphi ,k}^{g}\big\| _{e, {\mathcal {W}}^{(m)}_{\mu}/ {\mathbb {P}}_{m-1}\to {\mathcal {W}}^{(m+l-1)}_{\nu}/ {\mathbb {P}}_{m+l-2}} = \|gC_{\varphi } \|_{e, {\mathcal {W}}^{(m-k)}_{\mu}/ {\mathbb {P}}_{m-k-1}\to{\mathcal {W}}^{(m+l-k-1)}_{\nu}/ {\mathbb {P}}_{m+l-k-2}}. $$

### Proof

First we prove that

45 $$ \big\| C_{\varphi ,k}^{g}\big\| _{e, {\mathcal {W}}^{(m)}_{\mu}/ {\mathbb {P}}_{m-1}\to {\mathcal {W}}^{(m+l-1)}_{\nu}/ {\mathbb {P}}_{m+l-2}} \le\|gC_{\varphi }\| _{e, {\mathcal {W}}^{(m-k)}_{\mu}/ {\mathbb {P}}_{m-k-1}\to{\mathcal {W}}^{(m+l-k-1)}_{\nu}/ {\mathbb {P}}_{m+l-k-2}}. $$

Assume that $f\in{\mathcal {W}}^{(m-k)}_{\mu}/{\mathbb {P}}_{m-k-1}$. Recall that, since (31) and (32) hold, the operator $I_{k}$ boundedly maps space ${\mathcal {W}}^{(m-k)}_{\mu}/{\mathbb {P}}_{m-k-1}$ into ${\mathcal {W}}^{(m)}_{\mu}/{\mathbb {P}}_{m-1}$. Since equality (33) holds for every $h\in{\mathcal {W}}^{(m+l-1)}_{\nu}/{\mathbb {P}}_{m+l-2}$ and

46 $$ D^{j}\bigl(D^{k} h\bigr) (0)=0,\quad \overline {0,m+l-k-2}, $$

we see that $D^{k}:{\mathcal {W}}^{(m+l-1)}_{\nu}/{\mathbb {P}}_{m+l-2}\to{\mathcal {W}}^{(m+l-k-1)}_{\nu}/{\mathbb {P}}_{m+l-k-2}$. Moreover, we see that (34) holds.

Using these two facts and the boundedness of the operator $C_{\varphi ,k}^{g}:{\mathcal {W}}^{(m)}_{\mu}/{\mathbb {P}}_{m-1}\to {\mathcal {W}}^{(m+l-1)}_{\nu}/{\mathbb {P}}_{m+l-2}$, we see that the operator (21) maps the space ${\mathcal {W}}^{(m-k)}_{\mu}/{\mathbb {P}}_{m-k-1}$ to ${\mathcal {W}}^{(m+l-k-1)}/ {\mathbb {P}}_{m+l-k-2}$, and from (31), (34) and the boundedness of the operator $C_{\varphi ,k}^{g}:{\mathcal {W}}^{(m)}_{\mu}/{\mathbb {P}}_{m-1}\to{\mathcal {W}}^{(m+l-1)}_{\nu}/{\mathbb {P}}_{m+l-2}$, it follows that the operator $gC_{\varphi }:{\mathcal {W}}^{(m-k)}_{\mu}/{\mathbb {P}}_{m-k-1}\to{\mathcal {W}}^{(m+l-k-1)}/{\mathbb {P}}_{m+l-k-2}$ is also bounded. We also see that (22) holds for every $f\in{\mathcal {W}}^{(m)}_{\mu}/{\mathbb {P}}_{m-1}$.

Let $K:{\mathcal {W}}^{(m-k)}_{\mu}/{\mathbb {P}}_{m-k-1}\to{\mathcal {W}}^{(m+l-k-1)}_{\nu}/{\mathbb {P}}_{m+l-k-2}$ be a compact operator. Then the operator

$$\widetilde{K}:=I_{k}KD^{k}:{\mathcal {W}}^{(m)}_{\mu}/ {\mathbb {P}}_{m-1}\to{\mathcal {W}}^{(m+l-1)}_{\nu}/ {\mathbb {P}}_{m+l-2} $$

is compact.

Hence, as in (35) we have

47 $$ \big\| C_{\varphi ,k}^{g}f-\widetilde{K}f\big\| _{{\mathcal {W}}^{(m+l-1)}_{\nu}} \le \|gC_{\varphi }-K\|_{{\mathcal {W}}^{(m-k)}_{\mu}/{\mathbb {P}}_{m-k-1}\to {\mathcal {W}}^{(m+l-k-1)}/{\mathbb {P}}_{m+l-k-2}}\|f\|_{{\mathcal {W}}^{(m)}_{\mu}}. $$

By taking the supremum in (47) over the unit ball in ${\mathcal {W}}^{(m)}_{\mu}/{\mathbb {P}}_{m-1}$, and then taking the infimum in such obtained inequality over the set of all compact operators $K:{\mathcal {W}}^{(m-k)}_{\mu}/{\mathbb {P}}_{m-k-1}\to{\mathcal {W}}^{(m+l-k-1)}_{\nu}/{\mathbb {P}}_{m+l-k-2}$, we get (45).

Now we prove that

48 $$ \|gC_{\varphi }\|_{e, {\mathcal {W}}^{(m-k)}_{\mu}/ {\mathbb {P}}_{m-k-1}\to {\mathcal {W}}^{(m+l-k-1)}_{\nu}/ {\mathbb {P}}_{m+l-k-2}} \le \big\| C_{\varphi ,k}^{g}\big\| _{e, {\mathcal {W}}^{(m)}_{\mu}/ {\mathbb {P}}_{m-1}\to {\mathcal {W}}^{(m+l-1)}_{\nu}/ {\mathbb {P}}_{m+l-2}}. $$

Since (31) holds for every $f\in{\mathcal {W}}^{(m-k)}_{\mu}/{\mathbb {P}}_{m-k-1}$ and by using (32), we see that the operator $I_{k}:{\mathcal {W}}^{(m-k)}_{\mu}/{\mathbb {P}}_{m-k-1}\to{\mathcal {W}}^{(m)}_{\mu}/{\mathbb {P}}_{m-1}$ is bounded. From this, since $C_{\varphi ,k}^{g}:{\mathcal {W}}^{(m)}_{\mu}/{\mathbb {P}}_{m-1}\to{\mathcal {W}}^{(m+l-1)}_{\nu}/{\mathbb {P}}_{m+l-2}$ is bounded by the assumption, and $D^{k}:{\mathcal {W}}^{(m+l-1)}_{\nu}/{\mathbb {P}}_{m+l-2}\to{\mathcal {W}}^{(m+l-k-1)}_{\nu}/ {\mathbb {P}}_{m+l-k-2}$ is also bounded due to the inequality in (34) and (46), we see that the operator $D^{k}C_{\varphi ,k}^{g}I_{k}=gC_{\varphi }:{\mathcal {W}}^{(m-k)}_{\mu}/{\mathbb {P}}_{m-k-1}\to{\mathcal {W}}^{(m+l-k-1)}_{\nu}/{\mathbb {P}}_{m+l-k-2}$ is bounded. Beside this, note also that $C_{\varphi ,k}^{g}=I_{k}gC_{\varphi }D^{k}:{\mathcal {W}}^{(m)}_{\mu}/{\mathbb {P}}_{m-1}\to{\mathcal {W}}^{(m+l-1)}_{\nu}/{\mathbb {P}}_{m+l-2}$, where $D^{k}:{\mathcal {W}}^{(m)}_{\mu}/{\mathbb {P}}_{m-1}\to{\mathcal {W}}^{(m-k)}_{\nu}/{\mathbb {P}}_{m-k-1}$ and $I_{k}:{\mathcal {W}}^{(m+l-k-1)}_{\mu}/{\mathbb {P}}_{m+l-k-2}\to{\mathcal {W}}^{(m+l-1)}_{\nu}/{\mathbb {P}}_{m+l-2}$.

Let $K:{\mathcal {W}}^{(m)}_{\mu}/{\mathbb {P}}_{m-1}\to{\mathcal {W}}^{(m+l-1)}_{\nu}/{\mathbb {P}}_{m+l-2}$ be a compact operator. Then the operator

$$D^{k}KI_{k}:{\mathcal {W}}^{(m-k)}_{\mu}/ {\mathbb {P}}_{m-k-1}\to{\mathcal {W}}^{(m+l-k-1)}_{\nu}/ {\mathbb {P}}_{m+l-k-2} $$

is also compact.

Hence, as in (37) we have

49 $$ \big\| gC_{\varphi }f-D^{k}KI_{k}f\big\| _{{\mathcal {W}}^{(m+l-k-1)}_{\nu}} \le \big\| C_{\varphi ,k}^{g}-K\big\| _{{\mathcal {W}}^{(m)}_{\mu}/{\mathbb {P}}_{m-1}\to {\mathcal {W}}^{(m+l-1)}_{\nu}/{\mathbb {P}}_{m+l-2}}\|f\|_{{\mathcal {W}}^{(m-k)}_{\mu}}. $$

By taking the supremum in (49) over the unit ball in ${\mathcal {W}}^{(m-k)}_{\mu}/{\mathbb {P}}_{m-k-1}$, and then taking the infimum in such obtained inequality over the set of all compact operators $K:{\mathcal {W}}^{(m)}_{\mu}/{\mathbb {P}}_{m-1}\to{\mathcal {W}}^{(m+l-1)}_{\nu}/{\mathbb {P}}_{m+l-2}$, we get (48). From (45) and (48) is directly obtained (44), finishing the proof of the theorem. □
